# Well-Aligned TiO_2_ Nanotube Arrays with Ag Nanoparticles for Highly Efficient Detection of Fe^3+^ Ion

**DOI:** 10.1186/s11671-019-2883-4

**Published:** 2019-02-06

**Authors:** Zong-Min Ma, Xing-Sheng Wu, Dou-Dou Zheng, Jiu-Yan Wei, Yan-Na Xie, Yun-Bo Shi, Kun Huang, Xiao-Ming Zhang, Jun Liu

**Affiliations:** 1 Science and Technology on Electronic Test & Measurement Laboratory, North University of China, No.3, Xue Yuan Road, Taiyuan, 030051 Shanxi China; 2 School of Instrument and Electronics, North University of China, Taiyuan, 030051 China

**Keywords:** TiO_2_ nanotube arrays, Fe^3+^ detection, Functionalized Ag nanoparticle deposition

## Abstract

Nowadays, determination of the iron ions with high sensitivity and selectivity with novel methods becomes a matter of urgency for monitoring healthy body and environment. In this paper, for the first time, we present a set of high-performance TiO_2_ nanotube arrays which are quite sensitive to iron ions. Firstly, the anodic oxidation method was adopted to prepare ordered TiO_2_ nanotube arrays, followed by functionalized Ag nanoparticle deposition with the enhancement ability in iron ion sensing. Besides, the spectrum of the TiO_2_ nanotube with/without the Ag nanoparticles was analyzed with an X-ray photoelectron spectrometer, which shows that Ag nanoparticles can effectively reduce the recombination rate of electrons and holes, and increase the conductivity and the charge transfer rate of the electrodes. Further, when functionalized Ag nanoparticles on well-ordered TiO_2_ nanotube arrays were used, iron ion sensing performed with the anodic stripping voltammetry method was investigated to validate the great potential of TiO_2_ nanotube arrays with a sensitivity of approximately 30 μA/ug/L in becoming Fe^3+^ sensors. This method creates new possibilities for developing sensors for monitoring of Fe^3+^ in biological samples without any sample pretreatment procedure.

## Background

Iron is an important nutrient element for human health, which is usually responsible for transfer and transport of oxygen and block materials, and ranks second among the most essential metal elements in the human body. However, an excessive amount of iron ions in a living cell can catalyze production of reactive oxygen species (ROS) via the Fenton reaction, which can lead to diseases, such as kidney disease, and disturb the cellular homeostasis resulting in Alzheimer’s, Wilson’s, and Menkes diseases [1, 2]. As a matter of fact, due to its wide application in industry and agriculture, the potential toxic effects of iron from contaminated rivers, lakes, or oceans on humans remain a global challenge [3, 4]. Consequently, it is particularly necessary to monitor healthy body and environment to develop practical and efficient technologies used for rapidly determining the iron ions with high sensitivity and selectivity.

Nowadays, there are many methods for detecting metal ions, such as atomic absorption spectrometry, inductively coupled plasma mass spectrometry, and inductively coupled plasma emission spectrometry. However, complicated equipment and sample preparation steps are needed in these methods, which will also result in a large cost. Recently, an enormous effort has been made to studies on nanoparticles, such as graphene [5–12], graphene quantum dots [13–18], carbon dots [19–21], and noble metal nanoparticles or nanoclusters in the desired dimension for sensing of metal ions, which have attracted much attention because of good selectivity, high sensitivity, and easy operation. Vinod Kumar Gupta et al. have developed a method for synthesizing imine through a simple condensation reaction and exploring their metal detection abilities through electrochemical and optical methods. Fe^3+^ detected by colorimetric (L2) at a low concentration can reach 1.29 × 10^−6^ M. But this method is limited due to factors such as high detection limits, and strong interference [22]. Xiaohui Gao et al. reported a facile colorimetric sensor based on the N-acetyl-L-cysteine (NALC)-stabilized Ag nanoparticles (NALC–Ag NPs) for detection of Fe3+ ions in aqueous solution. This method can be used to perform sensitive and selective detection of Fe^3+^ ions in water with a linear range from 80 nM to 80 mM and a detection limit of 80 nM [23]. Kailasa S K et al. have developed a selective and sensitive colorimetric method for determination of Fe^3+^ ion by using p-amino salicylic acid dithiocarbamate functionalized gold nanoparticles (DTC-PAS-Au NPs) as colorimetric probes [24, 25]. On the basis of such detection, inductively coupled plasma (ICP) analysis coupled with mass spectrometry (MS) or optical emission spectroscopy (OES) was preferably adopted for iron ion analysis [26].

On the other hand, TiO_2_ nanotubes, with high chemical stability, are widely applied in various industries due to their excellent photo-electrochemical, catalytic, and adsorption properties as well as nontoxicity, such as gas/moisture sensors [27], photocatalytic decomposition of water into hydrogen [28], photocatalytic degradation of organic pollutants [29], dye-sensitized solar cells [30], biosensors [31], and supercapacitors [32]. Particularly, well-structured and highly ordered TiO_2_ nanotubes are well suitable for directional and rapid transfer of electrical charges [33–36]. Further, surface functionalization of Ag NPs plays a crucial role in increasing analytical applicability for TiO_2_ nanotubes detection of trace analytes with high selectivity and sensitivity. Hence, considering combination of TiO_2_ nanotubes and metal ion detection at trace level is highly desirable. As far as we know, there are very few reports on usage of functionalized TiO_2_ nanotubes as based probe for Fe^3+^ detection.

In this paper, a set of high-performance TiO_2_ nanotube arrays which are quite sensitive to iron ions are presented. Firstly, the anodic oxidation method was adopted to prepare ordered TiO_2_ nanotube arrays, followed by functionalized Ag NPs with the ability of iron ions sensing. Besides, the spectrum of the TiO_2_ nanotube with/without the Ag NPs was analyzed with an X-ray photoelectron spectrometer. Further, iron ion sensing with the use of functionalized Ag NPs on well-ordered TiO_2_ nanotube arrays through the anodic stripping voltammetry method was investigated to validate the great potential of heavy metal sensors of TiO_2_ nanotube arrays. This method creates new possibilities for developing sensors for monitoring Fe^3+^ in biological samples without any sample pretreatment procedure.

## Methods

### Principles of Sensing Iron Ions with TiO_2_

**Fig.1:**
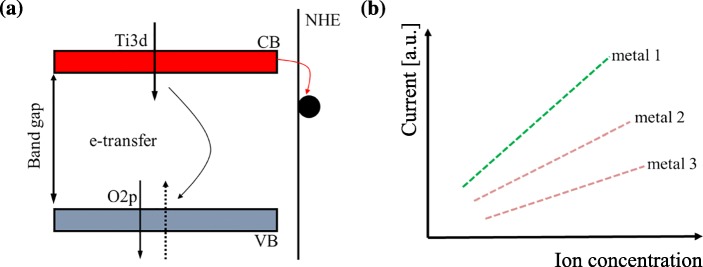
**a** The schematic diagram of the surface charge transfer between metal and TiO_2_. **b** Determination of concentration and species of metal ions based on the magnitude of the current induced by the charge transfer

The electrons will overcome the bandgap and transit from the valence band (VB) to the conductance band (CB) when absorbing enough energy in TiO_2_ nanotube. As a result, there will be a non-occupied electronic state (hole) in VB, which is positive, as explained in Fig. 1a. In this process, when an electron is excited from the VB to the CB, it can diffuse to the surface allowing charge transfer to an adsorbate or can become trapped within an electron trap in the band gap. If the surface of the nanotubes was covered by metal nanoparticles, charge transfer will occur between the adsorbate and the TiO_2_ nanotube because of electron transition. Based on this transfer, the amount of metal nanoparticles can be known when the quantity of electric charge is detected, as shown in Fig. 1b and Eqs. (1)–(3). From these equations, it can be seen that the excess charge distribution defines the potential that attracts the electron-withdrawing (O_2_) and repels the electron-donating (H_2_O) molecules to O_b_ vacancy defects when metal particles are adsorbed on the TiO_2_ surface. Therefore, we can detect the concentration and species of metal nanoparticles directly by measuring the amount of charge transfer with the following equations [37]:

1 $$ {\mathrm{Ti}}_{(6c)}\ \left[{(4s)}^2{(3d)}^2\ \mathrm{of}\ {\mathrm{Ti}}_{(6c)}\right]=4\times \left[{\mathrm{O}}_{\left(3\mathrm{C}\right)}\right]+2\times \left[{\mathrm{O}}_{\mathrm{b}\left(2\mathrm{C}\right)}\right] $$

2 $$ \kern1.5em \left[{(4s)}^2{(3d)}^2\ \mathrm{of}\ {\mathrm{Ti}}_{(6c)}\right]+\left[{\left(1\mathrm{s}\right)}^1\ \mathrm{of}\ \mathrm{H}\right]=4\times \left[{\mathrm{O}}_{\left(3\mathrm{C}\right)}\right]+1\times \left[{\mathrm{O}}_{\mathrm{b}\left(2\mathrm{C}\right)}\right]+\left[\mathrm{OH}\right]+{\mathrm{e}}^{-} $$

3 $$ {\mathrm{Ti}}_{(5c)}\ \left[{(4s)}^2{(3d)}^2\ \mathrm{electrons}\ \mathrm{of}\ {\mathrm{Ti}}_{(5c)}\right]=5\times \left[{\mathrm{O}}_{\left(3\mathrm{C}\right)}\right] $$

The stripping voltammetry method can be used to identify the concentration and species of metals on the surface of TiO_2_ nanotube arrays. In this method, the current’s dependence on the concentration and species of metals can be shown in Eq. (4) [38]:

4 $$ \Delta  {i}_p=\frac{nFA{D}^{1/2}\mathrm{C}}{\sqrt{\pi {t}_m}}\left(\frac{1-\sigma }{1+\sigma}\right) $$

Where, σ = exp[(*nF*/*RT*)(*∆E*/2)], and *N* is the number of electrons participating in reactions with metals. *F*, *A*, *D*, *C*, and *t*_m_ represent Faraday constant, area of the electrode, diffusion coefficient, and the concentration of the metal ions, and pulse width respectively. It can be seen from this equation that the current is proportional to metal concentration.

### Experimental Details

A pure titanium sheet (20 × 20 × 0.1 mm; purity 99.9%) was chemically polished to remove the oxide layer and create a smooth surface. To be specific, a proper amount of HF acid with a concentration of 40% was poured into ionized water proportionally (0.5%); then, the titanium sheet was dipped into the pre-prepared solution for 10 s and was taken out instantly and then dried with nitrogen. Next, it was ultrasonically washed in acetone, absolute ethyl alcohol, and deionized water at 30 °C for 12 min respectively. In the process, acetone was mainly used to clean the surface of the Ti sheet with a roughness of 0.139 μm and 0.066 μm before and after the treatment, respectively. Pretreatment of the Ti sheets is necessary prior to experiments for higher smoothness. The experimental setup was applied in Ref. [39]. The AMICUS X-ray photoelectron spectrometer (XPS) from Shimadzu was used for quantitative composition definition of the prepared TiO_2_ nanotube arrays.

Ag nanoparticles (NPs) with various sizes were deposited onto the TiO_2_ nanotubes surface (QPrep400, Mantis) with the power of 60 W under vacuum conditions of 10^−3^ Torr. Purity of the Ag plate was 99.9999%.

An electrochemical workstation (CHI660E, Shanghai Chenhua) was used to perform the stripping voltammetry experiments for detecting the concentration of Fe^3+^ ions. A three-electrode system (working, reference electrode, and auxiliary electrodes) which is consisted of two loops was used to monitor the electrochemical reaction process of the working electrode and to keep the equilibrium of the chemical reaction.

In the experiments, four types of Fe^3+^ with the concentrations of 10 μg/L, 20 μg /L, 30 μg/L, 40 μg/L, and 50 μg/L were mixed in ammonium chloride, respectively. When detected, neutral iron firstly was adsorbed onto the TiO_2_ nanotubes surface with/without Ag deposition after reduction reaction with Ti substrate, followed by re-oxidized when reverse bias was applied. The scanned voltage ranged from − 1 V to 1 V with a step of 0.005 V. The minimum detectable current was 10^−5^A, and the detected duration was 120 s. Concentration of Fe^3+^ ions was evaluated from the dissolution current peak’s dependence on voltage.

## Results and Discussion

### Preparation and Characterization of TiO_2_ Nanotube Arrays

**Fig. 2:**
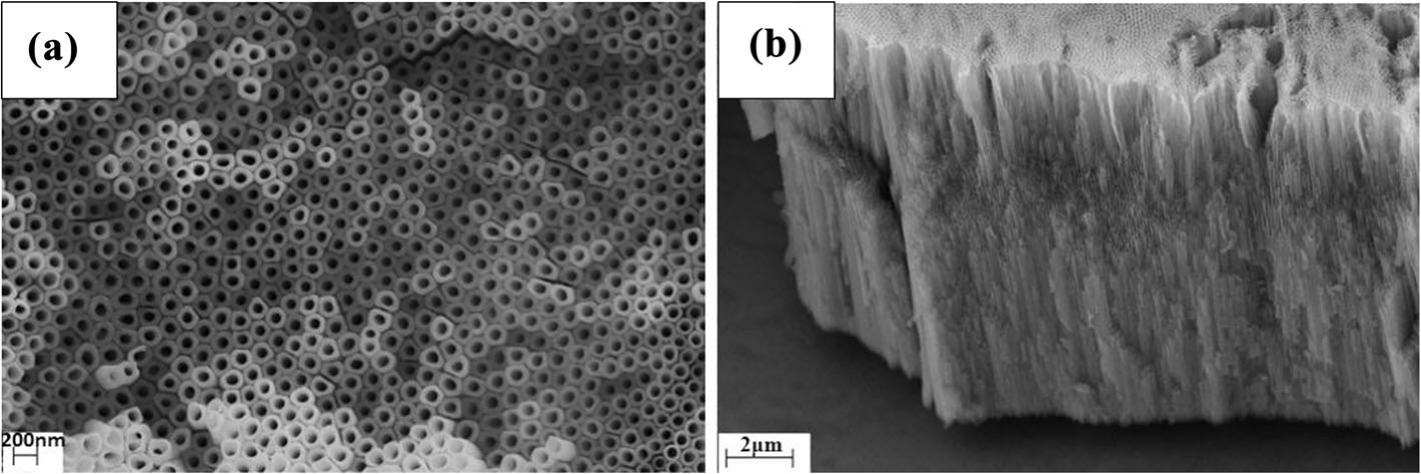
Topography (**a**) and Side-view (**b**) Images of the TiO_2_ Nanotube Arrays, respectively

TiO_2_ nanotube arrays produced in electrolyte composed of glycol, 2 vol% water and 0.3 wt% ammonium fluoride with the oxidation voltage being 60 V, the oxidation time being 2 h, the oxidation temperature being 40 °C, and the calcination temperature being 500 °C had the optimal morphology and performance [39]. Topography and side-view scanning electron microscope (SEM) images of the TiO_2_ nanotube arrays are shown in Fig. 2a, b, respectively. In Fig. 2a, the TiO_2_ nanotube arrays are well-arranged with an average diameter of 50 nm. In Fig. 2b, length of the nanotube is approximately 19.2 μm with a relative tilted angle of 30° with substrate when measured. From these results, it can be seen that the contact area with Fe^3+^ ions for the nanotube arrays can be obviously increased as length of the tube increases, which can improve detection sensitivity effectively.

**Fig. 3:**
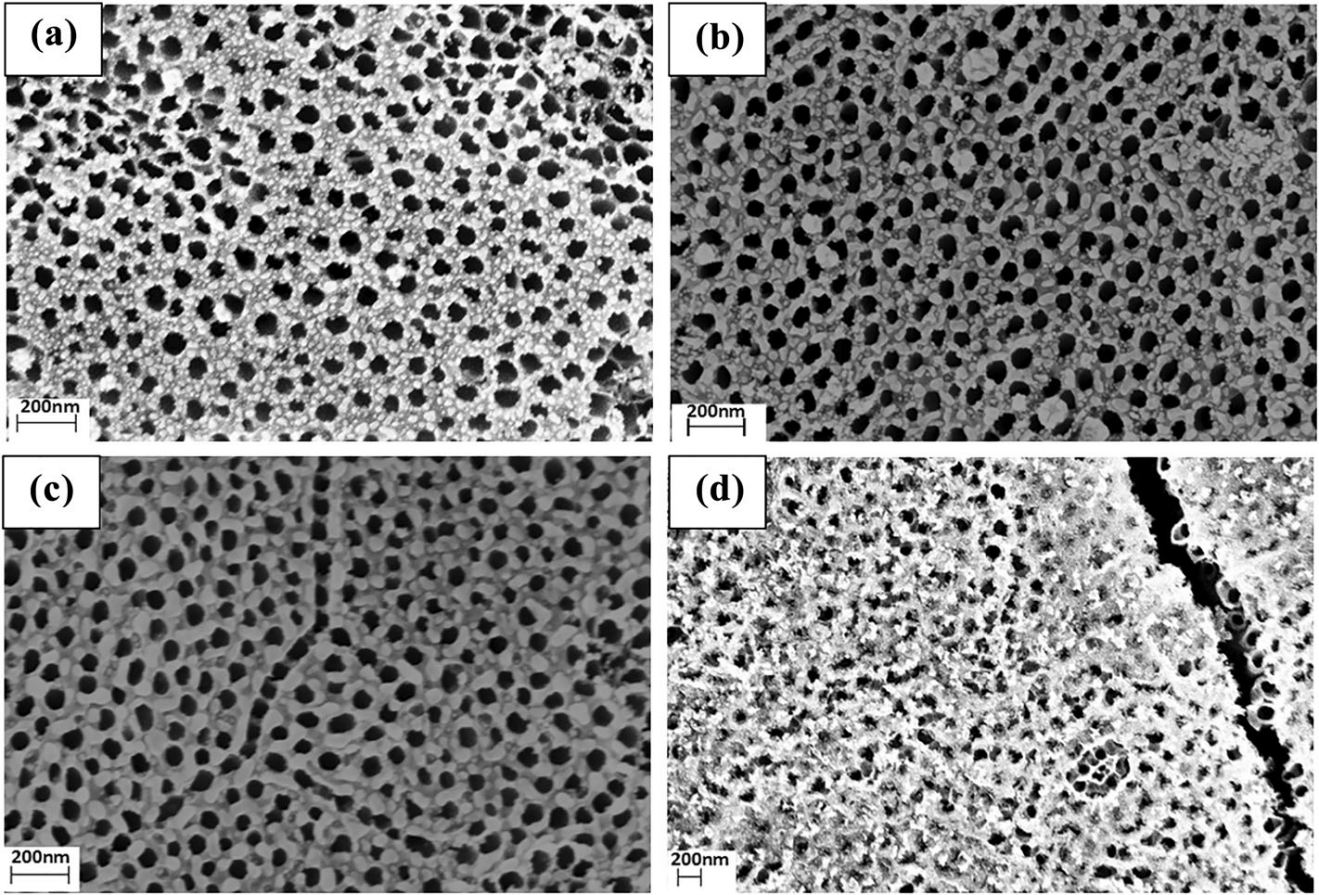
SEM Images of Ag Nanoparticle-loaded TiO_2_ Nanotube Arrays with sputtering durations of 30 s, 35 s, 40 s, and 45 s corresponding with (**a**) - (**d**), respectively

Figure 3a–d shows SEM images of TiO_2_ nanotubes with Ag nanoparticle deposition with durations of 30 s, 35 s, 40 s, and 45 s respectively. In Fig. 3a, when deposition duration was 30 s, the average diameter and coverage thickness of the Ag nanoparticles adsorbed on the wall of the nanotubes were approximately at 10 nm and 5 nm respectively. From these results, it can be seen that the nanoparticles are uniform in size distribution. In Fig. 3b, c, when deposition durations were extended to 35 s and 40 s, state of Ag nanoparticles became cluster state gradually and diameters of the Ag particles increased to 20 nm and 25 nm respectively. Diameters of the Ag nanoparticles further increased until they became clusters that partially cover the surface of the TiO_2_ nanotube when the deposition time increased to 45 s, which is shown in Fig. 3d.

**Fig. 4:**
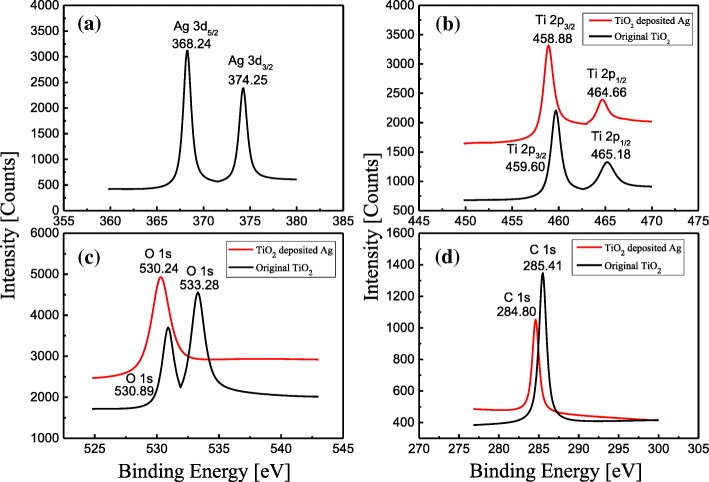
Photoelectron spectroscopy of the spectra of Ag in (**a**); and the spectroscopies of TiO_2_ Nanotubes with/without Ag Deposition in (**b**), (**c**) and (**d**) Measured by XPS, respectively

In order to confirm composition of the TiO_2_ nanotube arrays with/without Ag particle adsorption with a duration of 30 s, XPS experiments were performed, which are shown in Fig. 4a–d. In Fig. 4a, the spectra of Ag were given with the intensities of 3200 and 2400 counts when the values of binding energy were 368.24 eV and 374.25 eV, respectively, which showed that FWHM (full wave at half maximum) of Ag was approximately 1 eV. From this results, it can be seen that only one chemical state Ag^0^ was detected after Ag deposition.

In Fig. 4b, spectra of Ti with/without Ag deposition were given and marked with red and black lines. Before Ag deposition, the observed values of binding energy of Ti were 459.60 eV and 465.18 eV with the intensities being 2250 and 1250 counts, respectively. In contrast, the values of binding energy of Ti were 458.88 eV and 464.66 eV with the increasing intensities being 3250 and 2700 counts respectively after deposition. The difference in binding energy before Ag deposition was 5.58 eV; in contrast, the difference after deposition changed to 5.78 eV, which was increased by 0.3 eV approximately. Besides, the two titanium peak shifts were 0.72 eV and 0.52 eV respectively. This phenomenon was caused by the interaction of Ag NPs.

Similar phenomena were observed on the spectra for oxygen and carbon with/without Ag deposition, which are shown in Fig. 4c, d respectively. Before deposition, the values of binding energy of oxygen were 530.89 eV and 533.28 eV with the intensities being 3500 and 4500 counts, respectively. These results showed that the oxygen was in a negative two-valence state (lattice oxygen) and consisted of TiO_2_ with titanium. On the other hand, the value of binding energy of oxygen was 530.24 eV with the intensity being 4900 counts after deposition. Besides, the value of oxygen peak of binding energy, 533.28 eV, disappeared after deposition, and the oxygen peak shift was 0.67 eV. These results demonstrated that the state of oxygen was changed from lattice oxygen to adsorbed oxygen due to the reaction with Ag NPs after deposition.

In Fig. 4d, we detected the spectrum of carbon contamination on TiO_2_ nanotube arrays with/without Ag deposition, which were marked with red and black lines respectively. The value of binding energy of carbon contamination was 285.41 eV with the photon number intensity being approximately 1350 counts before deposition. On the contrary, the value of binding energy of carbon contamination was 284.80 eV with the photon number intensity being approximately 1050 counts after Ag deposition, which meant that the value of binding energy and the photon number of carbon was reduced by 0.59 eV and 30% respectively. These results indicated that the amount of carbon contamination was significantly reduced after Ag NPs deposition. In other words, deposition of Ag NPs can reduce contamination of the electrode as well as improve the efficiency of charge transfer. It should be noted that shift of binding energy measured after deposition was approximately 0.8 eV, which was mainly due to shift of carbon.

Therefore, TiO_2_ nanotube arrays deposited with Ag NPs can effectively reduce the recombination rate of electrons and holes, and increase conductivity and charge transfer rate of the electrodes compared to TiO_2_ nanotube arrays without Ag NPs.

### Sensing of Fe^3+^ Ions With/Without Ag NPs Deposition

**Fig. 5:**
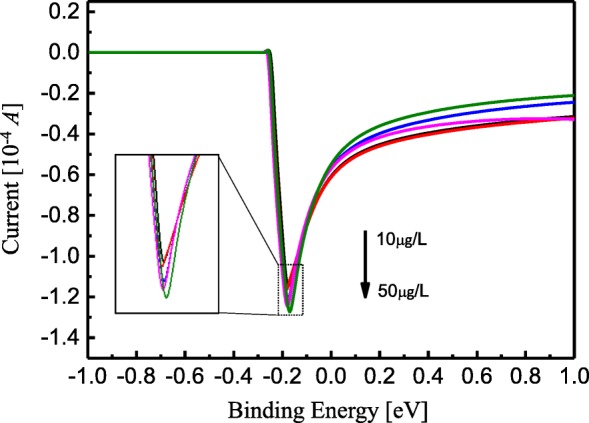
The dissolve current dependence of the voltage when detected Fe^3+^ ions

In order to obtain the experimental results, electrochemical stripping voltammetry was used to detect metallic Fe^3+^ ions with TiO_2_ nanotube arrays without Ag NPs deposition. The concentration of Fe^3+^ ions is set within a range from 10 μg/L to 50 μg/L. Dissolution current’s dependence on the voltage when Fe^3+^ ions were detected is shown in Fig. 5. During measurement, when the applied voltage was less than − 0.3 V, the dissolution current kept constant and remained approximately 0 A. When the applied voltage was within a range from − 0.3 V to − 0.16 V, the current dropped rapidly, and the peak reduced approximately from − 1.16 × 10^−4^ A to − 1.28 × 10^−4^ A with the Fe^3+^ ions concentration increment being within a range from 10 μg/L to 50 μg/L and with the voltage shifts being within a range from − 0.18 V to − 0.16 V, respectively. The inset shows current peak’s dependence on the applied voltage, as shown in Fig. 5. When the applied voltage V> − 0.16 V, the dissolution current increased exponentially and gradually, and stayed within a range from − 0.2 × 10^−4^ A to − 0.4 × 10^−4^ A. From this result, it can be seen that the peak of the current decreases with increase in Fe^3+^ ion concentration, and that the TiO_2_ nanotube array can be used as Fe^3+^ detection sensor.

**Fig. 6:**
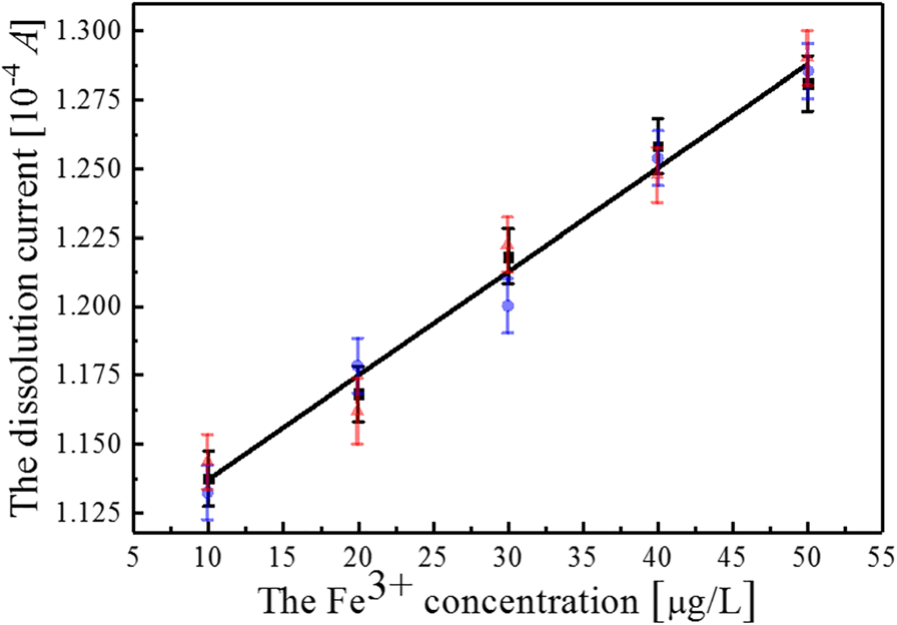
The current peaks dependence of the Fe^3+^ ions concentration

Dependence of the current peaks in Fig. 5 on Fe^3+^ ions concentration (from 10 μg/L to 50 μg/L) is also summarized in Fig. 6. The solid line is data fitting and is in alignment with linearity in a reasonable manner. Results of the fits can be calculated with *y* = 0.00373*x* + 1.1027, where *y* and *x* represent current and ion concentrations, respectively. This fitting result is sufficient for showing that the minimum detectable Fe^3+^ ion concentration when TiO_2_ nanotube arrays without Ag NPs deposition are adopted is 37.3 μA/μg/L. The detection limit of Fe^3+^ (*δx* = (*dy*/*dx*)^−1^·(1/*dy*)) when the TiO_2_ arrays are used should be 15.01 nM with consideration of linear range. Here, *dy*/*dx* = 37.3 μA/μg/L, and *δy* = 0.01 μA, *M*_Fe_ = 56, respectively. This result is comparable to that in Ref [25]. In this paper, the extinction ratio A700 nm/A520 nm is linear with the concentration of Fe^3+^ ranging from 40 mM to 80 mM, which can perform sensitive detection of Fe^3+^ ions with a detection limit of 14.82 nM when DTC-PASAu NP-based UV-visible method for on-site and real-time detection of Fe^3+^ in biological samples are adopted.

**Fig. 7:**
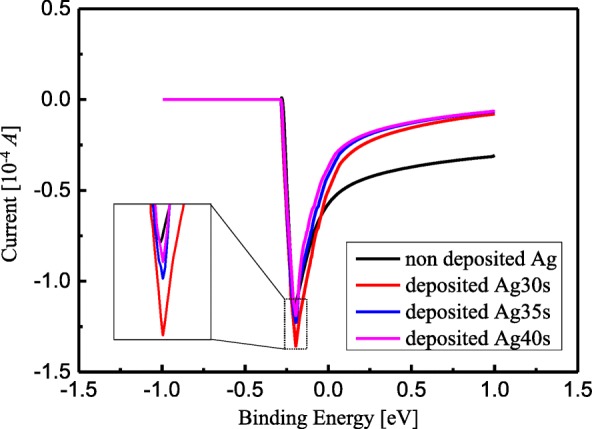
Detection of Fe^3+^ ions by Ag-loaded TiO_2_ nanotube arrays with various concentrations

**Fig. 8:**
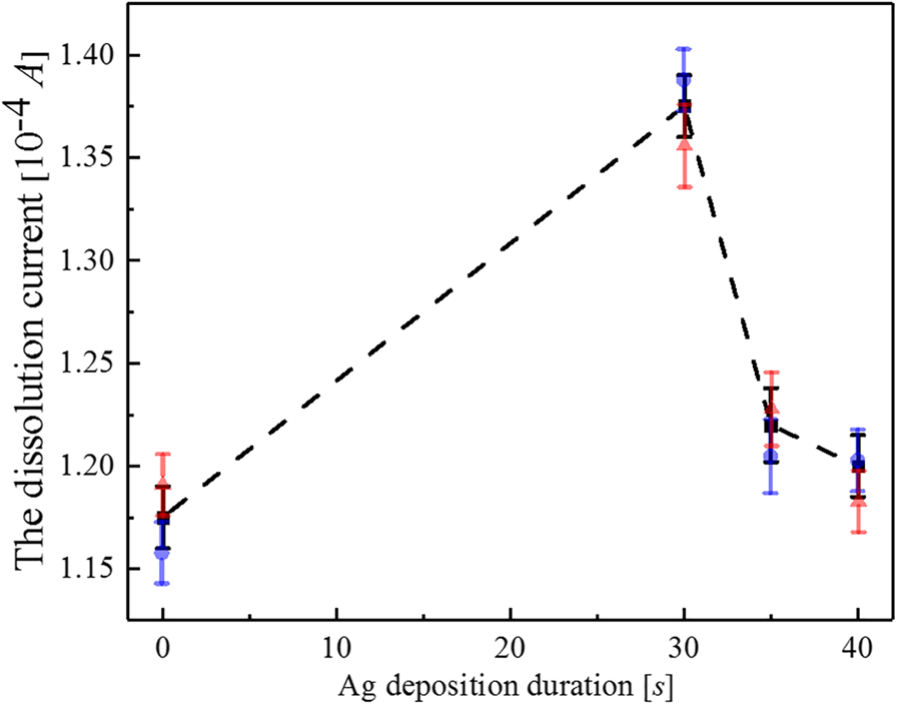
The relationship between the time of Ag ion deposition on TiO_2_ nanotube array and the detection concentration of Fe^3+^ ion

Next, sensitivity enhancement of Fe^3+^ concentration detection for TiO_2_ nanotube arrays with Ag deposition was investigated, with deposition durations being 30s, 35 s, and 40s respectively. The Fe^3+^ concentration was set at 10 μg/L, and the result is shown in Fig. 7. When the applied voltage V was less than − 0.3 V, the dissolution current kept almost constant and was approximately 0 A. When the applied voltage was within a range from − 0.3 V to − 0.2 V, the current dropped rapidly to approximately − 1.35 × 10^−4^ A, and when the applied voltage was greater than − 0.2 V, the dissolution current increased exponentially and gradually, and stayed within a range from − 0.08 × 10^−4^A to − 0.4 × 10^−4^ A. The inset shows current peak’s dependence on the applied voltage, as shown in Fig. 7. Dependence of the peaks in Fig. 7 on the Ag NPs deposition with the duration being within a range from 0 s to 40 s is also summarized in Fig. 8, and the solid line presented data fitting. It can be seen that the current reached the maximum value (approximately − 1.38 × 10^−4^ A) when the deposition time of Ag NPs was 30 s. In particular, compared to the value of current before Ag deposition onto TiO_2_ nanotube arrays, the value of current increased to approximately − 1.15 × 10^−4^ A, which meant that sensitivity of the Fe^3+^ detection was enhanced by 20% when Ag NPs deposition occurred on TiO_2_ surface. Besides, the maximum value of current in the deposition duration of 30 s was considered to be directly related with diameters and uniform distribution of Ag NPs on TiO_2_ surface. Consequently, TiO_2_ nanotube arrays are characterized by relatively simple fabrication, high sensitivity, and reproducibility compared to other Fe^3+^ detection methods. Moreover, color change of the Ag colloidal solution can be distinguished with naked eyes, which can also be used to directly check whether Fe^3+^ exists in the solution and serves as a strong basis for this research. Therefore, functionalized Ag NPs on well-ordered TiO_2_ nanotube arrays provided low-cost, high-selectivity, and sensitivity sensing response for Fe^3+^ with a low limit of detection under ambient conditions. Notably, enhancement of sensitivity of Fe^3+^ detection can also be realized for other Fe^3+^ solutions with different concentrations.

In order to demonstrate the selectivity of TiO_2_-Ag NPs for Fe^3+^ ions, competitive experiments were carried out in the presence of other metal ions when surface plasmon resonance (SPR), UV-visible spectrometry, etc. were used. Theoretically, the characteristic peak shift should occur only with the mixed solution of metal ions containing Fe^3+^ ions as indicated in Eqs. (1)–(4). We will focus on the competitive experiments next.

## Conclusions

TiO_2_ is an important functional material which is not only widely used in UV detectors, photocatalysts, and dye-sensitized solar cells, but also used in important potential applications in ultrasensitive sensors. In this paper, we present a method that utilizes well-aligned TiO_2_ nanotube arrays with Ag nanoparticles as voltammetry sensor for highly efficient detection of Fe^3+^ Ion. In the beginning, the anodic oxidation method was adopted to prepare ordered TiO_2_ nanotube arrays, followed by functionalized Ag NPs with the ability of iron ions sensing. Then, the spectrums of the TiO_2_ nanotube with/without the Ag NPs were compared by XPS, which proved that lattice oxygen in TiO_2_ nanotube arrays was released to adsorbed oxygen because of the interaction of Ag NPs. Therefore, when functionalized Ag NPs on nanotube arrays were used, iron ion sensing performed with the anodic stripping voltammetry method was investigated to validate their great potential in becoming heavy metal sensors, which proved that functionalized Ag NPs on well-ordered TiO_2_ nanotube arrays provided low-cost, high-selectivity, and sensitivity sensing response for Fe^3+^ with a low limit of detection under ambient conditions. This method creates new possibilities for developing sensors for monitoring of Fe^3+^ in biological samples without any sample pretreatment procedure.

## Abbreviations

CB: Conductance band
FWHM: Full wave at half maximum
NALC: N-acetyl-L-cysteine
NPs: Nanoparticles
ROS: Reactive oxygen species
SEM: Scanning electron microscope
Ti: Titanium
VB: Valence band
XPS: X-ray photoelectron spectrometer
